# Communication is crucial: Lessons from COVID‐19 vaccination and pregnancy

**DOI:** 10.1111/bcp.15578

**Published:** 2022-11-24

**Authors:** Christine Cole, Maria Tsakiroglou, Catriona Waitt

**Affiliations:** ^1^ Clinical Pharmacology and Therapeutics/Internal Medicine, Royal Liverpool University Hospital Liverpool University Hospitals NHS Foundation Trust Liverpool UK; ^2^ Institute of Systems, Molecular and Integrative Biology Department of Pharmacology and Therapeutics University of Liverpool Liverpool UK; ^3^ Department of Acute Medicine Liverpool University Hospitals NHS Foundation Trust Liverpool UK

**Keywords:** communication, Covid 19, pregnancy, vaccination, vaccine hesitancy

## Abstract

The morbidity and mortality from COVID‐19 infection are higher in pregnant women compared to their nonpregnant counterparts. As real‐world evidence accumulates demonstrating there is no increased risk of adverse maternal and neonatal outcomes associated with COVID‐19 vaccination during pregnancy, guidelines have evolved from a case‐by‐case benefit‐risk decision through to clear recommendation in April 2021 for COVID‐19 vaccination in pregnancy. However, vaccine hesitancy is a barrier to uptake, especially among the younger population and individuals of ethnic minority backgrounds; pregnant women have additional concerns. Trust in the importance and effectiveness of the vaccine, trust in public health agencies and science, together with good communication methods regarding the safety of COVID‐19 vaccines are strong factors for vaccination acceptance in pregnancy. Lack of trust in the health system was worsened by initial knowledge gaps in the information provided about COVID‐19 infection and the safety and immunogenicity of COVID‐19 vaccines. This was exacerbated by access to incorrect information and misinformation to fill in those knowledge gaps, especially with the increased use of social media. To provide advice and reassurance on COVID‐19 vaccine safety to pregnant women, healthcare professionals involved in their care should have the knowledge and skills to provide risk‐benefit communication and would benefit from access to training in science communication. Clinical pharmacologists have the expertise to appraise and synthesize emerging pharmacovigilance data, which can inform and support risk‐benefit communication by other clinicians. Information should be strategically directed at individual audiences, taking their perspectives and foundational belief systems into consideration.

The COVID‐19 pandemic has cost the lives of over six million people worldwide,^1^ and there is now substantial evidence that morbidity and mortality are higher in pregnant women^2^, ^3^, ^4^, ^5^ compared to nonpregnant women. Furthermore, adverse birth outcomes, including preterm birth, low birth weight and stillbirth, are significantly higher following maternal COVID‐19, especially when maternal illness has been severe.^6^ In the face of this, the fight for interventions to combat both the spread of and mortality associated with the disease has been relentless. COVID‐19 is the clinical disease resulting from infection with severe acute respiratory syndrome‐related coronavirus 2 (SARS‐CoV‐2), therefore in addition to therapeutics for the management of mild to severe COVID‐19 that have been licensed for inpatient and outpatient use, vaccines are a mainstay in this war. As of May 2022, 10 vaccines have been granted approval for emergency use.^7^

The administration of vaccines to pregnant women has its own complexities. Of note, the majority of pregnant women take medicines that are not licensed for use in pregnancy due to their exclusion from clinical trials; evidence of the safety of medications in pregnancy is primarily derived from postmarket surveillance data.^8^ For ethical reasons, therapeutics under trial are rarely tested in this cohort due to concerns about teratogenicity and fetotoxicity, and until pre‐clinical developmental and toxicity reproductive data become available it may be unjustifiable to include pregnant women in a clinical trial. However, the blanket exclusion of pregnant people from clinical trials is sometimes counterproductive and has resulted in the inequity in access to important medications, including vaccines for pregnant individuals.^9^ This is because evidence‐based clinical outcomes for commonly used therapeutics only become available in this population whilst being used in clinical practice where greater numbers of women are exposed to these drugs.^10^ Lessons learned from the rubella vaccination programme in the United States and the ebola virus vaccination programme in Central Africa show that systematic exclusion of pregnant women from vaccination programmes due to concerns of safety may actually increase their risk(s) associated with the disease, a phenomenon that has been described as being “protected to death”.^11^, ^12^ It has been proposed that the balance between safety concerns of fetotoxicity versus equitable access to therapeutics in pregnant women can be achieved with a stepwise approach taking into consideration preclinical animal reproductive data and information from nonpregnant women enrolled in trials, including opportunistic studies among women who become pregnant whilst taking part in a clinical trial.^13^ When vaccines against SARS‐CoV‐2 were first approved, there was no evidence on their safety profile in pregnant women (as well as the foetus and neonates) due to their exclusion from vaccine trials,^14^, ^15^ and the general advice with regards to COVID‐19 vaccination was to make a case‐by‐case risk‐benefit decision for each pregnant individual.^15^ However, as it became evident that maternal and foetal outcomes are significantly worse in those infected with the SARS‐CoV‐2 virus^16^ as well as growing evidence of the efficacy and safety of COVID‐19 vaccines in pregnant women and lactating mothers from preclinical studies and accumulation of observational data, the risk‐benefit considerations now overwhelmingly support the use of COVID‐19 vaccination in pregnancy (see Figures 1 and 2). Ongoing postauthorization surveillance of COVID‐19 vaccine use in pregnant women has enabled further characterization of vaccine safety in pregnant women to be undertaken and confirm that covid vaccination during pregnancy produces a good immunological response with no increased risk in adverse maternal and neonatal outcomes. Evidence to support the efficacy and safety of COVID‐19 vaccination in pregnancy is summarized in Tables 1 and 2, respectively. The United Kingdom national surveillance and safety analysis of COVID‐19 vaccination in pregnancy outlines the modalities in place to monitor the safety of COVID‐19 vaccines in pregnancy, including surveillance and reporting systems, such as the United Kingdom Teratology Information Service (UKTIS), the United Kingdom Obstetric Surveillance System (UKOSS) and UK surveillance of vaccination in pregnancy (VIP).^16^ UKOSS data derived between March 2000 and March 2022 will enable assessment of the outcomes of COVID‐19 infection in pregnancy for both mothers and their infants.^23^ British guidelines have changed in the light of the clear evidence indicating that the benefits of vaccination clearly outweigh any potential risks, to actively encourage COVID‐19 vaccination in pregnancy. The Royal College of Obstetricians and Gynaecologists recommends COVID‐19 vaccination for pregnant women across all three trimesters.^24^ The European Medicines Agency (EMA), based on a review of studies undertaken by the COVID‐19 task force involving 65 000 pregnancies, reports that mRNA COVID‐19 vaccines did not increase the risk of pregnancy complications or compromise foetal wellbeing.^25^ They further affirm that COVID‐19 vaccines reduce the risk of hospitalizations and deaths in pregnant women as in the nonpregnant population.^25^ Counterparts in the United States also recommend COVID‐19 vaccination in pregnant women and lactating mothers.^26^, ^27^

**FIGURE 1:**
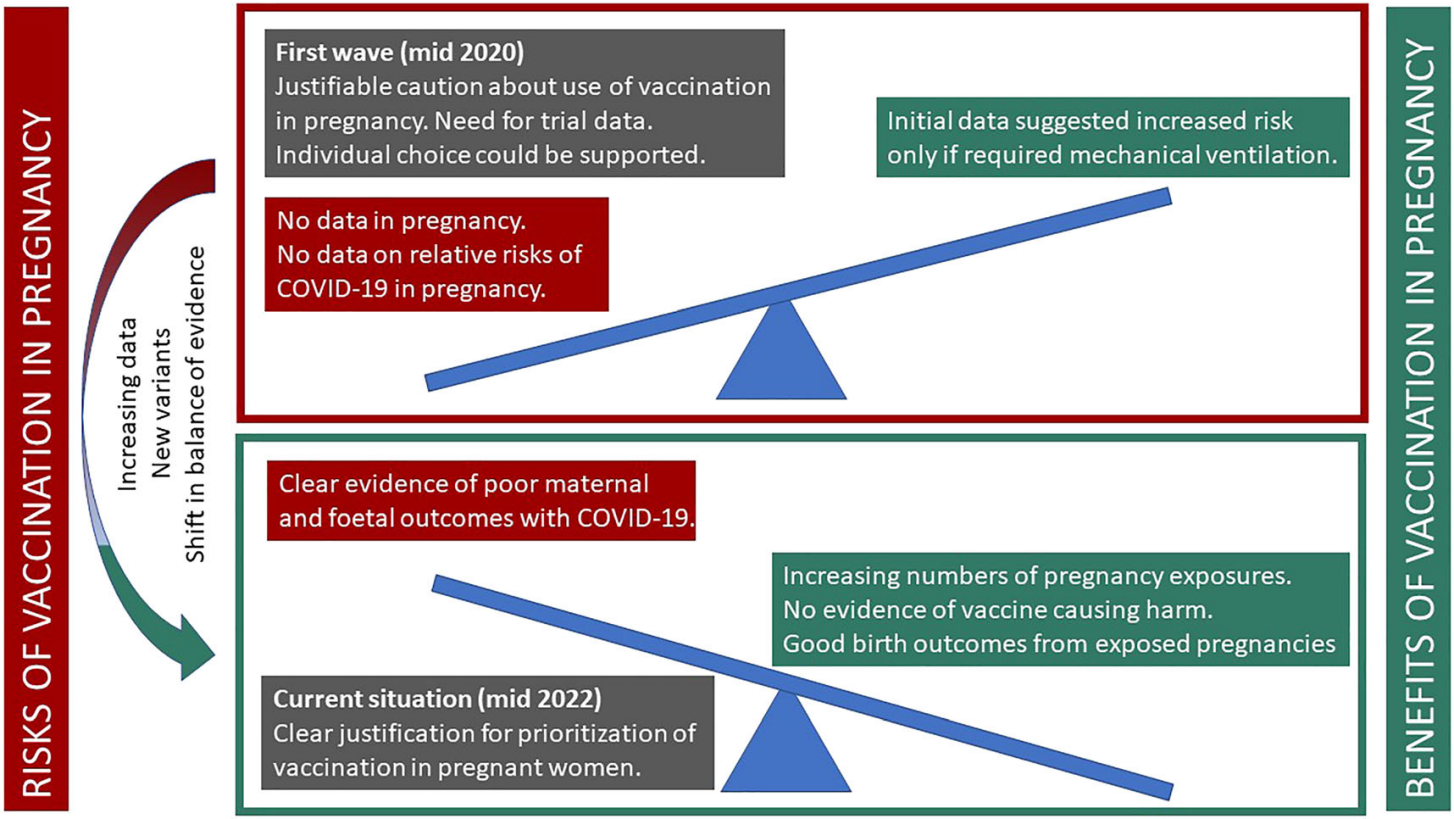
Changing risk‐benefit ratio of COVID‐19 vaccination in pregnancy with emerging evidence

**FIGURE 2:**
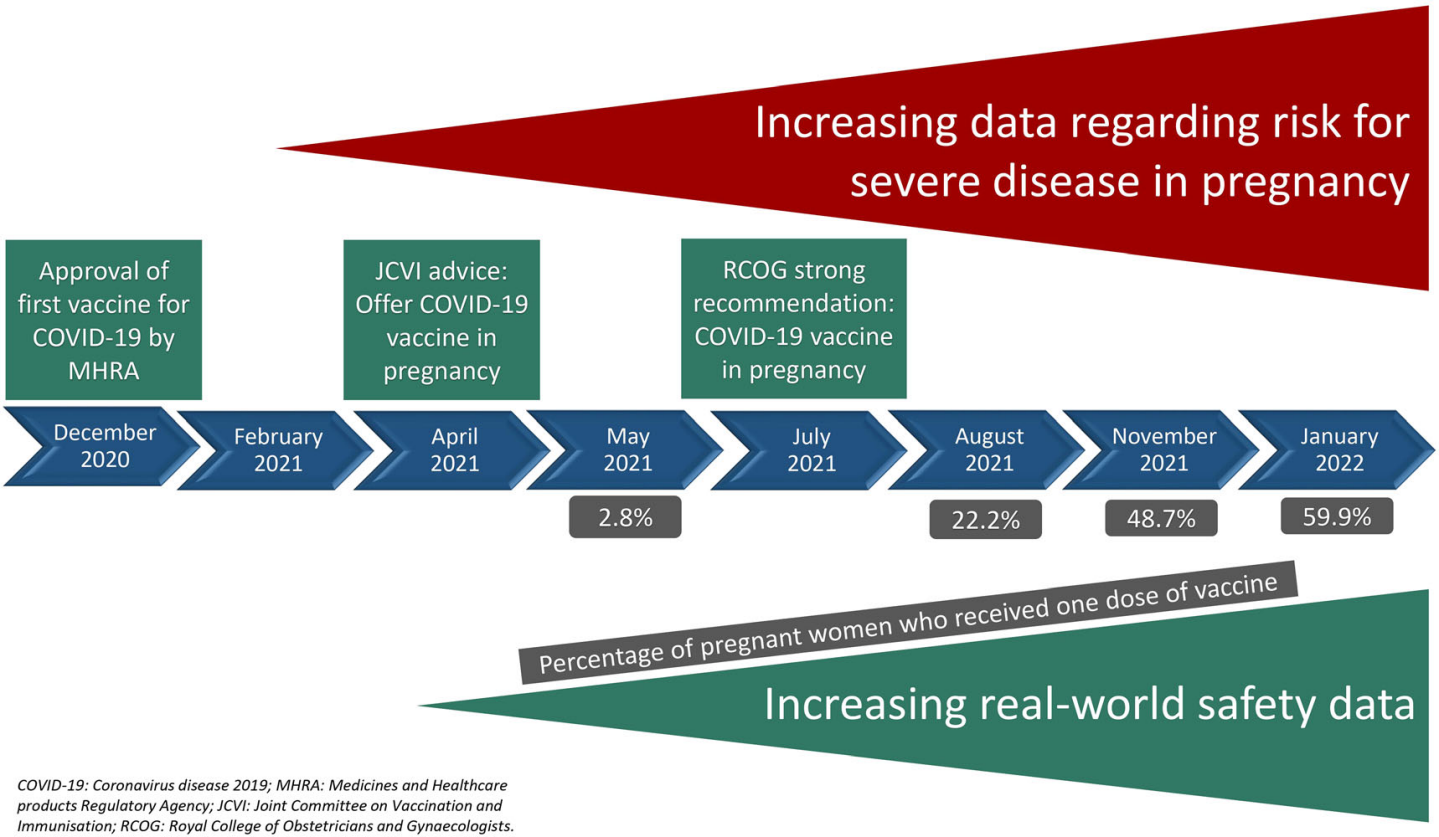
COVID‐19 vaccination in pregnancy: summary of important timelines

**TABLE 1:** Summary of systematic reviews of safety outcomes for COVID‐19 vaccination in pregnant women

Study	Study design	Vaccine type	Material outcomes	Neonatal outcomes	Adverse events
COVID‐19 Vaccination among pregnant people in the US: A systematic review^17^ Published 10 March 2022	Search conducted between 1 January 2020 and 6 February 2022 22 studies (5 cohort, 10 cross‐sectional, 4 case reports, 1 pre‐post, 1 case‐control and 1 case series	PfizerBioNTech BNT162B2 Moderna mRna‐1273 Jannsen	No statistical differences in pregnancy outcomes such as gestational hypertension (*P* = .60), pre‐eclampsia (*P* = 1.00) and thromboembolism incidence (*P* = 1.00) between vaccinated and unvaccinated pregnant women. No placental injuries or stillbirths. Miscarriage rates after receiving COVID‐19 vaccination ranged from 6.50% to 14.10%. These rates were similar to the 11‐16% expected rate of miscarriage in the general population.	No increased risk of adverse neonatal outcomes due to COVID‐19 vaccination during pregnancy. No neonatal deaths were reported. Other neonatal outcomes, including preterm birth (9.40%, 5.90%), congenital anomalies (2.20%, 1.20%), small size for gestational age (3.20%, 12.20%) and NICU admission (0.70%, 15.30%) following COVID‐19 vaccination were similar to the expected rate of neonatal outcomes in the unvaccinated population.	Side‐effects reported in pregnant women were similar to the general population. The most common side‐effects were injection‐site pain and soreness, fevers or chills, fatigue and itching.
SARS‐CoV‐2 vaccines during pregnancy and breastfeeding: A systematic review of maternal and neonatal outcomes^18^ Published 5 March 2022	Search conducted up to 27 October 2021. 46 studies included in the qualitative synthesis. 32 studies included pregnant women (a global cohort of 74 908 pregnant women and 5082 lactating women who received COVID‐19 vaccination)	PfizerBioNTech BNT162b2 Moderna mRNA‐1273 Oxford‐AstraZeneca ChAdOx1 Jannsen	10 studies evaluated safety outcomes such as spontaneous abortion and pregnancy loss, postpartum haemorrhage and caesarean delivery. In a propensity score‐matched cohort, the rates of adverse pregnancy outcomes were similar to that of unvaccinated pregnant women. Vaccination did not increase the rate of pregnancy complications.	No neonatal deaths in three studies that reported neonatal safety outcomes. In one study two (3.5%) babies needed NICU admission. COVID‐19 vaccination during the third trimester lowered the risk of adverse neonatal outcomes.	No additional adverse events of vaccination during pregnancy compared to non‐pregnant women. Rate and severity of adverse effects were unaffected by the timing of vaccination during pregnancy. In one study, 68/7,530 women vaccinated during pregnancy (0.9%) reported possible vaccine‐related adverse events and none were serious. Headache (0.1%), overall asthenia (0.1%), unspecified pain (<0.1%), and stomachache (<0.1%) were the most often reported side‐effects.
Effectiveness and safety of COVID‐19 vaccine among pregnant women in real‐world studies: A systematic review and meta‐analysis^19^ Published 6 February 2022	Search conducted for studies from inception of vaccination to 6 November 2021. 6 studies (observational cohort studies) included in a meta‐analysis of adverse pregnancy outcomes among 40 978 pregnant women with and without COVID‐19 vaccination (19 108 vaccinated and 21 870 unvaccinated).	PfizerBioNTech BNT162b2 PfizerBioNTech BNT162b2 + Moderna mRNA + adenovirus vector vaccine	Postpartum haemorrhage with blood transfusion: vaccinated 14/273, unvaccinated 41/2261, OR 1.181(0.625‐2.234) Spontaneous vaginal delivery: vaccinated 160/273, unvaccinated 1459/2261, OR 0.899 (0.690‐1.171) Operative vaginal delivery: vaccinated 28/273, unvaccinated 111/2261, OR 1.514 (0.955‐2.401) Caesarean delivery: vaccinated 85/273, unvaccinated 691/2261, OR 0.979 (0.741‐1.293) Eclampsia or preeclampsia up to 72 h from delivery: vaccinated 21/7670, unvaccinated 44/9392, OR 0.912 (0.507‐1.640) Gestational hypertension: vaccinated 19/140, unvaccinated 225/2,862, OR 1.142 (0.691‐1) Thromboembolism within 4 weeks before or after delivery: vaccinated 0/129, unvaccinated 2/1580, OR 2.438 (0.116‐51.047)	5‐minute Apgar score <7: vaccinated 3/140, unvaccinated 38/1862, OR 1.051 (0.320‐3.449)	Not assessed
mRNA Covid‐19 vaccines in pregnancy: A systematic review^20^ Published 2 February 2022	Search conducted 20‐22 June 2021; one article added on 13 July 2021 13 observational studies with a total of 48 039 pregnant women who received mRNA vaccines	PfizerBioNTech BNT162b2 Moderna mRNA‐1273	No statistically significant differences in pregnancy outcomes such as eclampsia/pre‐eclampsia (*P* = 1), gestational hypertension (*P* = .6038), gestational age (*P* = .7028) and incidence of thromboembolism (*P* = 1) between vaccinated and unvaccinated groups; abortion rate and preterm birth 12.6% (104/827) and 9.4% (60/636), respectively Statistically, vaccination did not affect delivery outcomes such as birth trauma (*P* = 1), uterine rupture (*P* = 1), unplanned ICU admission (*P* = .1956), blood loss >1000 mL (*P* = .4452), haemorrhage with transfusion (*P* = .3531) and mode of delivery (*P* = .6517) Other outcomes reported among vaccinated and unvaccinated pregnant women were abortion (1.7% [128/7530] *vs* 1.6% [118/7530]) and preeclampsia (0.3% [20/7530] *vs* 0.3% [21/7530])	The effects of mRNA vaccines on neonatal outcomes were reported by four observational studies. 15% (2/13) of cases required NICU admission, 8% (1/13) experienced TTN and 8% (1/13) required supplemental oxygen or CPAP. Neonatal outcomes were compared between vaccinated and unvaccinated pregnant women: intrauterine growth restriction (0.5% [36/7530] *vs* 0.5% [38/7530]), stillbirth (1/7530 [<0.1] *vs* 2/7530 [<0.1]) and preterm birth (<37 weeks) (77/1387 [5.6%] *vs* 85/1427 [6.0%]). NICU admission (*P* = .5821), Apgar score at 5 min <7 (*P* = .7617), hypoxic ischemic encephalopathy (*P* = 1), stillbirth (*P* = 1) and low birth weight (*P* = .5321) or very low birth weight (*P* = .2332) did not differ significantly between vaccinated and unvaccinated women.	Injection site pain was the most common local adverse event Fatigue, myalgia headache, chills, fever and nausea were the most common systemic adverse events. Seizure was reported in a woman who received the mRNA vaccine but this patient had a history of seizure disorder and the anticonvulsant level in the blood was borderline low.
Adenovirus‐based vaccines and thrombosis in pregnancy: A systematic review and meta‐analysis^21^ Published 2 February 2022	Search conducted for studies between 1 January 1966 and 9 August 2021 167 studies included in the systematic review	Adenovirus vaccines	From trials where vaccine arm allocation and pregnancy outcomes were reported, 80 (57.5%) pregnancies in the adenovirus vector vaccine group resulted in healthy full‐term births and 76 (61.8%) pregnancies in the placebo or nonadenovirus vector arms resulted in healthy full‐term births. Consistent with the established background risk of major birth defects of 2‐4% and miscarriage of 15‐20% in the general population of the United States No cases of DVT, cerebral sinus thrombosis, or other coagulopathy was reported with these pregnancies. 12 studies were included in the meta‐analysis. Using a fixed‐effects model (*I* ^2^ = 0%, *P* > .05), the RR of adverse events in pregnancy was reported as 0.675 (0.448‐1.017). Across all pregnancy events, the RR for pregnancy was not different between adenovirus and non‐adenovirus arms. No increased risk of thrombocytopenia, coagulopathy or adverse birth outcome associated with receipt of adenovirus vaccination in pregnant women.	Not assessed	Not assessed
COVID‐19 vaccination in pregnant and lactating women: A systematic review^22^ Published 11 October 2021	Results up to 12 June 2021 10 studies included	PfizerBioNTech BNT162b2 Moderna mRNA‐1273	104 miscarriages out of 827 women who had a complete pregnancy and 65 (1.52%) preterm deliveries out of 636 pregnant women The rate of abortions was not different from nonvaccinated pregnant women studied before the COVID‐19 pandemic	6 (0.14%) NICU admissions	Not assessed

Abbreviations: CPAP, continuous positive airway pressure; DVT, deep venous thrombosis; NICU, neonatal intensive care unit; OR, odds ratio; RR relative risk; TTN, transient tachypnea of the newborn.

**TABLE 2:** Summary of systematic reviews of efficacy outcomes for COVID‐19 vaccination in pregnant women

Study title	Vaccine	Maternal efficacy outcomes	Neonatal immunogenicity outcomes
COVID‐19 vaccination among pregnant people in the US: A systematic review^17^	PfizerBioNTech BNT162b2 Moderna mRNA‐1273 Jannsen	10 of 32 studies in pregnant women examined the immunogenicity of the COVID‐19 vaccine. COVID‐19 vaccination during pregnancy produced a good immune response and antibody production was similar to that of nonpregnant women. Immunity produced by the COVID‐19 vaccination was significantly stronger than that obtained after natural infection with the virus (*P* < .05). Vaccination was effective in preventing COVID‐19 infection among pregnant women and significantly reduced the risk of future COVID‐19 infection (*P* < .05) in vaccinated pregnant women compared to unvaccinated pregnant women. 9 of 2136 (0.40%) and 3 of 1822 (0.20%) pregnant women experienced COVID‐19 infection >14 days after the first Pfizer‐BioNTech and Moderna vaccine, respectively.	Anti‐SARS‐CoV‐2 antibodies were found in umbilical cord blood meaning that COVID‐19 vaccination during pregnancy may convey some immunity to neonates
SARS‐CoV‐2 vaccines during pregnancy and breastfeeding: A systematic review of maternal and neonatal outcomes^18^	PfizerBioNTech BNT162b2 Moderna mRNA‐1273 Oxford‐AstraZeneca ChAdOx1 Jannsen	22 studies assessed immunogenicity post COVID‐19 vaccination. Following maternal vaccination, there was a good maternal immune response and transfer of maternal antibodies to confer passive protection against SARS‐CoV‐2 in newborns.	The existence of anti‐SARS‐CoV‐2 antibodies in breast milk suggests a possible specific protective effect on the newborn‐infant after both maternal infection and vaccination
mRNA Covid‐19 vaccines in pregnancy: A systematic review^19^	PfizerBioNTech BNT162b2 Moderna mRNA‐1273	Among pregnant women who received the Pfizer‐BioNTech vaccine, 0.1% (3/2136) and 0.4% (9/2136) had COVID‐19 infection within 14 days of vaccination and more than 14 days after vaccination, respectively Among pregnant women who received the Moderna vaccine, 0.4% (7/1822) and 2% (3/1822) had COVID‐19 infection within 14 days of vaccination and more than 14 days after vaccination, respectively Vaccination significantly reduced the risk of future infection (*P* = .0004) and all cases of infection reported in the first trimester among vaccinated women occurred prior to the first vaccination. There was no significant reduction of the risk of COVID‐19 infection within 10 days from vaccination (*P* = .79), but risk reduction reached statistical significance 11‐27 days after vaccination (*P* < .001), and 28 days or more after vaccination (*P* < .001). Vaccination induced immunoglobulin (Ig)G and IgM production in 71% (87/122) of pregnant women; 16% (19/122) of pregnant women produced only IgG, while in 13% (16/122) neither IgG nor IgM was detectable Vaccination provided a rapid immunological response after the first dose, while infection provided a gradual immunological response	IgG was detected in 89% (25/28) of cord blood, but no cases had detectable IgM
COVID‐19 vaccination in pregnant and lactating women: A systematic review^22^	PfizerBioNTech BNT162b2 Moderna mRNA‐1273	Positive maternal antibodies were present in 334/351 pregnant women and positive umbilical cord blood antibodies in 221/351 pregnant women 16 women did not have detectable antibodies at birth; all of them delivered within 4 weeks after first‐dose administration	Not assessed

Against this backdrop, efforts to achieve vaccination have been hampered by scepticism towards the COVID‐19 disease itself as well as towards vaccines. This has been fuelled by conspiracy theories on social media and other media platforms which became a major means of connecting people when restrictions for physical interaction with others were in place. The problem of “vaccine hesitancy” was highlighted as one of the major threats to global health by the World Health Organization even before the current pandemic and has been defined as “the delay in acceptance or refusal of vaccines despite availability of vaccine services”.^28^ It encompasses a wide continuum between complete acceptance and complete refusal^29^; it is important for clinicians to consider this when communicating with their patients and the public, so that an individual's genuine concern(s) can be gently addressed. It is understood that psychological factors including complacency (not perceiving diseases as high risk and vaccination as necessary), convenience (practical barriers) and confidence (lack of trust in safety and effectiveness of vaccines) influence hesitancy.^29^ In the United Kingdom, psychosocial factors (trust, concerns and beliefs) and communications were more common barriers to vaccination in ethnic minorities than practical and logistical factors.^30^ In the United Kingdom and the United States, vaccine hesitancy is associated with ethnic minorities (particularly among black/African American communities), female sex, younger age and lower socioeconomic status.^31^, ^32^, ^33^ Concerns about the safety of the COVID‐19 vaccines, especially with regards to long‐term side effects, is a major factor affecting uptake in ethnic minority groups^30^, ^34^; the speed of COVID‐19 vaccines development and approval, as well as the inadequate representation of subjects from racial and ethnic minorities in vaccine clinical trials and clinical trials in general for various reasons^30^, ^35^, ^36^ have influenced this. In addition, a general mistrust in the healthcare system is another major factor affecting COVID‐19 vaccine uptake in ethnic minorities.^34^ The history of negative past experiences with formal services (reported as experiencing discrimination and neglect from medical professionals when accessing healthcare) and institutional racism in the healthcare system have impacted on the health decisions made by people from ethnic minority backgrounds in the United Kingdom and the United States.^30^, ^33^, ^34^, ^37^ Razai et al^34^ cited systemic racism and discrimination, previous unethical healthcare research in black populations, under‐representation of minorities in health research and vaccine trials, and negative experiences within a culturally insensitive healthcare system as factors that cause mistrust, producing vaccine hesitancy. People who reported such negative experiences are more likely to be hesitant to vaccination, including the COVID‐19 vaccine.^33^, ^38^ In the United States in particular, previous unethical healthcare research in the black ethnic group has been documented to influence COVID‐19 vaccine hesitancy.^38^ With regards to communication, people from ethnic minorities were more likely to have received misinformation about the vaccine encouraging them not to take it; such information was frequently derived from sources other than mainstream media such as social media and word of mouth. The decision to take a vaccine was also influenced by family members to a large extent. Conflicting and changing guidance, together with contradiction of information between different information sources caused confusion and increased vaccine hesitancy. In addition, positive information provided by the government or National Health Service (NHS) added to the perception that the risks associated with the vaccines were not being fully communicated.^30^ Due to poor health literacy, language barriers and increased digitalization of communication, many people in this population could not access the information provided by reliable sources due to lack of access or knowledge of technology.^30^ Lack of trust in the health system and public authorities has been compounded by initial knowledge gaps (such as duration of immunity and need/rationale for repeat vaccinations) in the information provided about COVID‐19 and the rapidly changing evidence consequently leading to changes in the information provided as more was being understood about the disease. Misinformation therefore easily spreads to fill in the knowledge gaps.^39^

Historically, pregnant women have been concerned about taking vaccinations, not only for their health, but more importantly for the safety of their babies,^40^ possibly due to insufficient pregnancy safety data to inform their decisions. Moreover, there is evidence of scepticism and hesitancy among pregnant women with regards to COVID‐19 vaccination. COVID‐19 vaccine uptake was consistently lower for each month in Scotland between December 2020 to October 2021 in pregnant women than in the general female population of 18‐44 years. In October 2021, 42.8% (*n* = 1738) of all women who delivered (*n* = 4064) had received any COVID‐19 vaccine, with only 32.3% having received two doses. This is compared to 84.7% (*n* = 803 241) of women aged 18‐44 years in the general population (*n* = 947 984) having received any vaccination with 77.4% having received two doses by October 2021.^41^ Most of those who become pregnant fall into the younger age bracket, who are known to be more likely to be hesitant towards taking the COVID‐19 vaccine. Concerns about safety and insufficient information about the vaccine and side effects were the most common reasons for vaccine hesitancy given by 456 pregnant and postpartum women in Ohio, United States.^42^ A systematic review on the perspectives of pregnant women to receiving COVID‐19 vaccination by Januszek et al^43^ concludes that appreciating the severity of the disease and safety of the vaccine as well as provision of trustworthy information about the need for and safety of vaccines are the strongest factors for vaccine acceptance. Trust in the importance and effectiveness of the vaccine, trust in public health agencies and science, having accepted previous vaccinations, as well as good communication methods on the safety of COVID‐19 vaccines are strong factors for accepting COVID‐19 vaccination by pregnant women. Older age, higher education and higher socioeconomic status were also positive contributory factors.^43^

A more positive trend in COVID‐19 vaccine uptake in pregnancy has been noted from statistics between August and November 2021. In October 2021, 41.3% of women giving birth in England had received at least one dose of vaccine compared to 22.5% in August 2021. These improvements were noted across demographics: 13.3% of pregnant black women had a first dose of a COVID‐19 vaccine by delivery, up from 5.5% in November 2021. However, vaccine coverage is still low in pregnant women of black ethnicity and in pregnant women living in deprived areas of England compared to the general cohort of pregnant women in the United Kingdom^44^ and this is similar in other developed countries. Results from a study in the United States report that black pregnant women are less likely to take the vaccine than white pregnant women due to higher uncertainty about the long‐term effects of the COVID‐19 vaccine; “knowing a pregnant friend who received it” was the most common answer in black participants as to what will reduce their concerns about the vaccine, compared to “published data” being the most common answer in white participants.^45^ In addition to knowing someone who had taken the vaccine, previous influenza vaccination and being concerned about the risk of COVID‐19 to personal wellbeing and that of the baby are other enabling factors for COVID‐19 vaccination in pregnant women.^42^

Information provided by health professionals and scientists is generally regarded by the public to be credible.^46^ The American Society for Reproductive Medicine suggests ways whereby healthcare workers can support members of the public to make decisions about vaccines,^47^ which include personal experience sharing, emphasizing the stringent standards in vaccine development whilst reinforcing accurate information and also providing reliable sources of information about vaccination.^47^ Bond and Nolan's study of 45 Australian parents provides insight into the concept that making choices about vaccination for their children involves their perception of risk versus benefit. Although the population under discussion is different, the authors make salient points about risk communication for vaccination. It is important to note that communication of risk should be individualized; providing general information that portrays equality of risk may be suboptimal because perceptions of what comprise acceptable versus unacceptable risk of contracting a disease, as well as perceptions of severity of the disease vary between individuals^48^ and possibly between communities as well.

Ensuring transparency in vaccine communication, including the anticipated and emerging adverse event profiles, is important so the public can be assured they are making an evidence‐based risk‐benefit decision when receiving vaccines. Clinical pharmacologists are in a unique position to appraise and assimilate emerging evidence on the safety of vaccines, with emphasis on the role of pharmacovigilance and postmarket surveillance. Their in‐depth understanding and involvement in all aspects of drug development and regulation as well as the fact that patient safety is at the cornerstone of all clinical pharmacology efforts puts risk‐benefit communication within the purview of clinical pharmacologists. This synthesis of evidence can inform concise summaries and support communications for healthcare professionals with direct patient‐facing roles who may have less familiarity with pharmacovigilance methodology. Communicating the risks and benefits of vaccines is especially pertinent for healthcare providers from ethnic minority backgrounds. It has been shown that provision of information about the vaccine from someone patients can identify with as a trusted source, such as healthcare workers from different ethnic backgrounds, promotes vaccine acceptance among ethnic minorities.^30^ However, as put quite aptly by Paulik et al,^49^ “In risk communication, trust carries more weight than technical credentials”. The public's trust in recommendations from public health and government authorities is a significant factor in receiving information about the COVID‐19 pandemic and vaccines.^49^ Among the black and Asian ethnic minority community those who do not have a negative opinion about whether public officials genuinely care about their wellbeing are more willing to take vaccines.^31^ Messages should be strategically directed at individual audiences, taking their perspectives into consideration and their foundational belief systems. The complexity of tailoring a technical message to a nontechnical audience should also be considered.^49^ The public receive pandemic health risk communication through a number of sources, such as official government websites and traditional mainstream media, but also from informal sources such as friends, relatives, health professionals and social media. An individual's viewpoint is formed by the information received from all these sources. Younger people are more inclined to trust information received from informal sources.^46^ However, there is under‐representation of information from credible official sources such as the World Health Organization on social media platforms. For instance, on the world's largest video‐sharing platform, YouTube, 25% of the most popular videos related to COVID‐19, which elicited more than 62 million views worldwide, contained misleading content and governmental videos that contained factual and accurate information accounted for only 11% of the videos and 10% of the views.^46^ During the pandemic, messages that included videos were shared more frequently than those without.^46^

To provide adequate advice and reassurance on COVID‐19 vaccine safety in pregnancy, healthcare professionals involved in their care should have the required knowledge and expertise to provide effective risk‐benefit communication. The importance of communication skills training for all healthcare workers engaged in providing services to pregnant people cannot be overemphasized. Communications training of healthcare personnel who are trusted sources of healthcare information for minority ethnic groups should include strategies to initiate discussions about vaccinations and tailoring conversations to address vaccine beliefs has been recommended.^33^ The NHS is in a unique position to provide on the job training in communication skills to health professionals as it employs the majority of medical staff in the United Kingdom. Communication messages should be strategically directed at individual audiences, taking their perspectives and foundational belief systems into consideration. Specifically, clinical pharmacologists are a small group of accredited physicians that have the knowledge, experience and expertise to assimilate the emerging safety and efficacy data and present it in a simple, accessible format; this can support the risk‐benefit communications by many healthcare workers. However, formal training in science communications and public engagement is not currently provided to medical trainees in the UK national postgraduate curriculum, and we are not aware of structured training in this area internationally. This should be an area for consideration by either incorporating communications into curricula or leveraging on partnership with the British Pharmacological Society and Royal Colleges to prove ad hoc public engagement and communications training as needed. Public engagement and involvement are recognized as an essential component of clinical research by several British funders, including the National Institute for Health and Care Research, The Wellcome Trust and the Medical Research Council; it is important that universities and other research organizations recognize the importance of outputs relating to public engagement and science communication as well as the more traditional metrics of conference presentations and peer‐reviewed scientific papers. To improve communication, governments in collaboration with professional bodies and stakeholder organizations should ensure timely dissemination of information to health workers and that all information being provided is consistent to avoid conflicting messages^33^; such information should be continuously reviewed, aligned and amended as new evidence and practical details emerge.^33^ This is because proactive, clear and open communication provided in real time enhances institutional trust.^50^ The Royal College of Midwives (RCOM) and Royal College of Gynaecologists (RCOG) provide clear information and advice for pregnant women and health professionals on COVID‐19 vaccination on their respective websites.^51^, ^52^ The key communication lessons learned from the COVID‐19 pandemic are summarized in Figure 3. These lessons will pave the way for further proactive engagement with the public. Furthermore, we can also learn from pandemic‐prone regions of the world and how they have surmounted communication barriers to provide valuable health messages to their communities.

**FIGURE 3:**
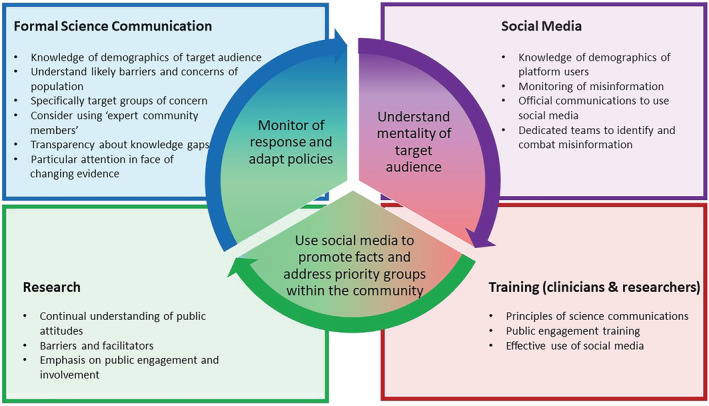
Key communication lessons from the COVID‐19 pandemic

## COMPETING INTEREST

There are no competing interests to declare.

## Data Availability

Data sharing is not applicable to this article as no new data were created or analysed in this study.
